# CXCL10 Produced by HPV-Positive Cervical Cancer Cells Stimulates Exosomal PDL1 Expression by Fibroblasts *via* CXCR3 and JAK-STAT Pathways

**DOI:** 10.3389/fonc.2021.629350

**Published:** 2021-08-06

**Authors:** Xiaona Chen, Hui He, Yue Xiao, Ayshamgul Hasim, Jianlin Yuan, Min Ye, Xin Li, Yi Hao, Xia Guo

**Affiliations:** ^1^Center for Clinical Research and Innovation (CCRI), Shenzhen Hospital, Southern Medical University, The Third School of Clinical Medicine, Southern Medical University, Shenzhen, China; ^2^Clinical Medical Research Center, Shenzhen Hospital, Southern Medical University, Shenzhen, China; ^3^Department of Pathology, Shenzhen Hospital, The University of Hong Kong, Shenzhen, China; ^4^Department of Pathology, Basic College, Xinjiang Medical University, Urumqi, China; ^5^Department of Gynecology, Affiliated Cancer Hospital, Xinjiang Medical University, Urumqi, China; ^6^Department of Pathology, Affiliated Cancer Hospital, Xinjiang Medical University, Urumqi, China; ^7^Department of Ultrasound, South China Hospital of Shenzhen University, Shenzhen, China

**Keywords:** human papillary virus, CXCL10-CXCR3 axis, innate and adaptive immune responses pathways, TLR signaling pathways, PD-L1, cervical cancer, JAK-STAT

## Abstract

Persistent infection with human papillomavirus (HPV) and immune surveillance failure may be the initiating factors for the carcinogenesis of cervical squamous cell carcinoma (CSCC). HPV infection might affect the innate immune pathway of cervical epithelial cells that constitute the “microenvironment” for tumor cells. Programmed death-ligand 1 (PD-L1) has been reported to be an immunosuppressor that helps cancer cells escape the actions of T cells. In the present study, CXCL10 was substantially upregulated both in cervical tissues of HPV infected patients with cervical intraepithelial neoplasia (CIN) or CSCC, as well as in HPV16 E6/E7 transgenic murine cervix. The HPV-positive (HPV+) cervical cancer cell lines SiHa and Caski secreted increased levels of CXCL10 compared to human foreskin fibroblasts (HFF-1), and its receptor CXCR3 was overexpressed in HFF-1. After co-culture with SiHa or Caski, the JAK-STAT signaling pathway and exosomal PD-L1 expression were both upregulated in HFF-1. Recombinant human CXCL10 induced JAK-STAT and PD-L1, while the CXCL10-CXCR3 and JAK-STAT inhibitors AMG487 or ruxolitinib reduced the expression of PD-L1 in HFF-1 cells. Furthermore, the upregulated expression of PD-L1 was verified in HPV+ but not HPV-negative (HPV-) patients with cervical cancers by analysis of tissue microarray cores in 25 cervical lesion patients (*P* < 0.05). The results indicate that HPV infection can induce cervical cancer cells to secrete CXCL10, which binds to CXCR3 in the surrounding fibroblast cells,leading to JAK-STAT pathway activation and the subsequent upregulated expression of exosomal PD-L1. These mechanisms may help HPV to escape immune response attack, leading to carcinogenesis.

**Graphical Abstract:**
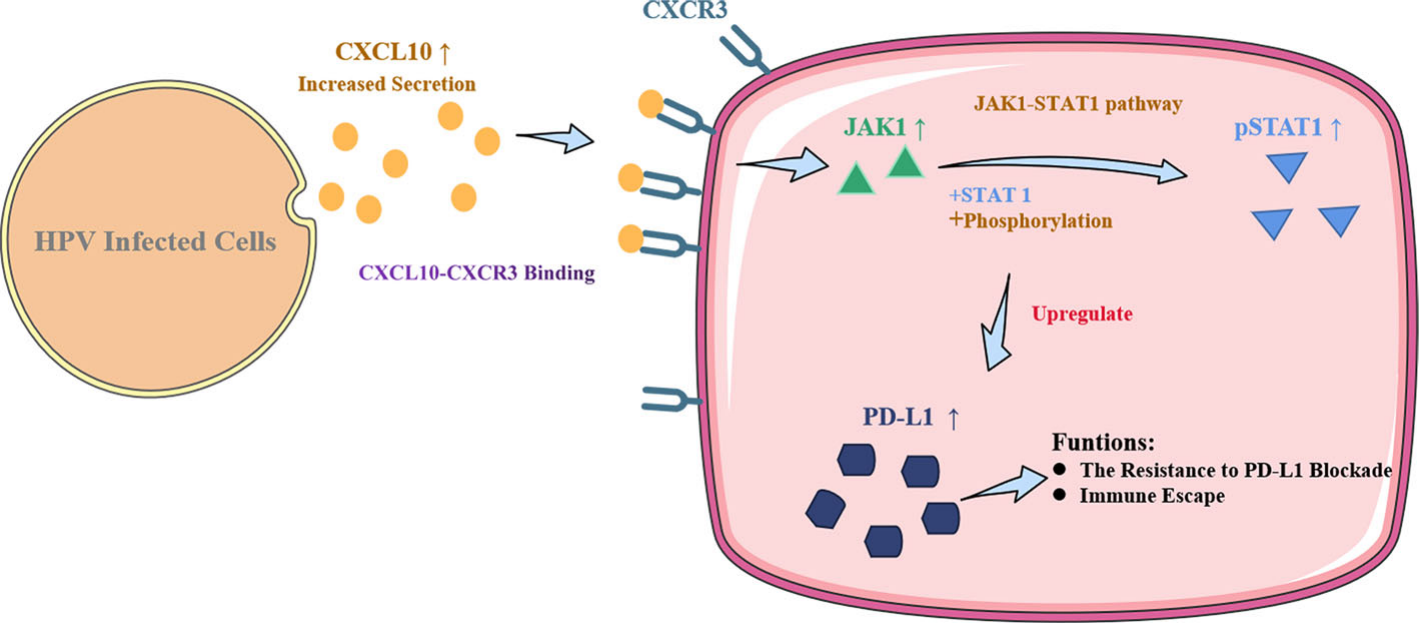


## Introduction

Cervical cancer is the second most common malignancy in women after breast cancer, and a high-risk of human papillomavirus (hrHPV) infection is the primary cause (1, 2). While most HPV infections can be cleared by the human immune system, a small subset of persistent infections develop from cervical intraepithelial neoplasia I (CINI) that progresses to CIN III and then invasive carcinoma (3, 4). Therefore, persistent infection with hrHPV and immune surveillance failure may be the initiating factors for cervical cancer. The HPV E6 and E7 oncogenes (5), key factors in viral carcinogenesis, are capable of causing malignant transformation by targeting the critical tumor suppressors p53 and pRb, which are effectively the “Achilles heel” of genomic stability (6, 7). However, less is known about how HPV genes help HPV-infected cells to evade immune responses (8, 9). Viruses have developed powerful immunosuppressive mechanisms including targeting antigen processing and presentation, which are the processes required for effective innate and adaptive immune responses (10).

Innate immunity is the body’s first line of defense against microbial and virus invasion. Previous studies have demonstrated that signals from a virus invading cells can be recognized by sentinel receptors in the natural immune system, which in turn triggers the activation of innate immune pathways, regulate adaptive immune responses and initiate downstream signaling cascades that produce proinflammatory cytokines and chemokines (11, 12). CXC motif chemokine 10 (CXCL10), also known as interferon gamma-induced protein-10 (IP-10), is a small cytokine-like protein secreted by a wide variety of cell types. CXCL10 is a ligand of the CXC chemokine receptor-3 (CXCR3) and is predominantly expressed by T helper cells (Th cells), cytotoxic T lymphocytes (CTLs), dendritic cells, macrophages, natural killer cells (NKs), as well as some epithelial and cancer cells (13, 14). The CXCL10/CXCR3 signaling pathway mediates paracrine interactions between tumor and stromal cells that govern leukocyte trafficking and angiogenesis. Emerging data have implicated the non-canonical CXCL10/CXCR3 axis in tumorigenesis and metastasis.

Recent research has suggested that the CXCL10/CXCR3 axis may be associated with the promotion and induction of various types of cancers including breast cancer (15, 16), pancreatic cancer(PCC) (17), hepatocellular carcinoma (HCC) (18, 19), melanoma (17), lymphoma (20), brain cancer (21), papillary thyroid carcinoma (PTC) (22), lung adenocarcinoma (23) and osteosarcoma (24). In the present study, CXCL10 expression was substantially upregulated both in cervical tissues of HPV infected patients with CIN or CSCC, as well as in hrHPV16E6/E7 transgenic mice, as determined by PCR array analysis of gene expression profiling in innate and adaptive immune response pathways; these findings suggested a pivotal role for hrHPV in driving tumorigenesis. Cytotoxic T lymphocyte dysfunction is frequently associated with activation of PD-L1/PD-1 and is a principal obstacle in opposing cancer therapy. Several studies have investigated whether HPV infection can affect PD-L1 expression in cervical cancer and found that HPV-positivity was positively correlated with increased PD-L1 expression (25). In the present study, the mechanisms underlying HPV E6/E7 induced evasion of cervical cancer cells from the host immune system *via* the CXCL10-CXCR3 axis were investigated (26).

## Methods

### Collection of Clinical Specimens

#### Tissue Samples

Cervical lesions tissues and matched cervical exfoliated cell specimens were collected from cervical lesion patients, including normal tissue (20 cases, NC), high grade squamous intraepithelial lesion (HSIL, including CIN II/III lesions), and early cervical squamous cell carcinoma (staged before IIa, 20 cases, CSCC) at the Affiliated Tumor Hospital of Xinjiang Medical University with patients’ agreement. These cases were selected according to clear pathological diagnoses based on the World Health Organization (27) diagnostic criteria. The patients had not previously received preoperative anticancer therapy. Informed consent was obtained from each patient enrolled and the collection of tissue specimens was approved by the Internal Review and Ethics Boards of the Affiliated Tumor Hospital of Xinjiang Medical University. Tissue microarray chips containing 90 pairs of CSCC tissue specimens, matched to adjacent non-tumoral (NT) cervical tissue specimens, and the associated clinicopathological information were purchased from Shanghai OUTDO Biotech Co. (Shanghai).

### Detection and Genotyping of HPV

The Hybrid Capture^®^ 2 system (hc2, Digene Corp., USA) was purchased by the Cancer Hospital affiliated to Xinjiang Medical University. The second-generation hybrid capture 2 (hc2) assay is the only method currently approved for clinical hrHPV DNA testing by the US Food and Drug Administration (FDA) and the China Food and Drug Administration (CFDA). A total of 37 HPV genotypes including 16, 18, 31, 33, 35, 39, 45, 51, 52, 56, 58, 59, 66, 68, 6, 11, 42, 43, 44, 81, 53, 26, 34, 40, 54, 55, 57, 61, 67, 69, 70, 71, 72, 73, 82, 83 and 84, and multiple infections could be accurately detected with both a sensitivity and specificity > 95%. Especially, based on the guidelines used for clinical diagnosis, the method can detect 13 HR-HPV types (-16, -18, -31, -33, -35, -39, -45, -51, -52, -56, -58, -59 and -68) or 5 LR-types (-6, -11, -42, -43 and -44), as well as the HPV DNA load. The assay was a non-radioactive signal-amplification method based on the hybridization of the target HPV-DNA to labeled RNA probes in solution, utilizing signal amplification of antibody capture and detection of chemiluminescence signals in 96-well plates.

### PCR Array Analysis of Innate and Adaptive Immune Responses as Well as TLR Signaling Pathways

Two groups (HSIL, CSCC) of HPV16/18-positive patients with cervical lesions (a total of 31 cases) and the cervical tissue specimens without HPV16/18 lesions or other HPV subtypes (NC) as the controls were selected from 60 specimens previously collected. RT^2^ profiler PCR array gene expression analysis was used for differential analysis of TLR signaling pathways (QIAGEN, Cat. no. PAHS- 018Z), as well as innate and adaptive immune response pathways (84 genes) (QIAGEN, Cat. no. PAHS-052Z) (**Tables S2A, B**) by using the human tissue sample described above. PCR arrays were used to analyze the differential expression of receptors, virus-specific response molecules, pathway regulatory molecules, upstream and downstream effector molecules in innate and adaptive immune response, Th1, Th2, Th17, Treg marker molecules, inflammatory response and anti-viral immune response molecules. The operational steps were performed according to Qiagen’s operating procedures. The protocol can be briefly described as follows: Total RNA was extracted from tissue using a RNeasy Microarray Tissue Mini Kit and purified (RNeasy@ MinElut™, Qiagen) to ensure RNA quality. Reverse transcriptase MLV-RT was used to synthesize the cDNA using 1.0 µg of total RNA as a template. 2 x SYBR Green Fluor qPCR Master Mix was prepared according to the requirements of the assay. Subsequently, sample cDNA and MasterMix were added to 96 wells, respectively in which 84 genes, 5 housekeeping genes and 7 controls were detected. Online data analysis software was used to analyze the original CT values obtained by real-time quantitative PCR to form a scatter map, and the RNasy microarray tissue mini-kit map was grouped.

### Cell Culture

HFF-1, and Caski cells were purchased from the Cell Bank of Chinese Academy of Sciences. H8 and SiHa cells were kindly provided by Prof. Abulizi Abudula. SiHa, Caski and H8 cells were cultured in RPIM1640 media (Gibco, US) and HFF-1 cultured in DMEM (HyClone, US) supplemented with 10% fatal bovine serum (Biological Industries, Israel), and 100 U/ml penicillin/streptomycin (Biological Industries, Israel). Cells were cultured in a 5% CO_2_ incubator at 37°C. When cultures became confluent, the cells were treated with 0.25% trypsin (Gibco, US) containing 0.02% EDTA for 2 min at room temperature.

### HPV E6/E7 Knockout by CRISPR/Cas9 Gene Editing

CRISPR/Cas9 single guide sequences specifically targeting HPV16 infection marker E6/E7 (sg E6/E7) were designed in Vector Builder (www.vectorbuilder.com) and produced by the Cyagen Company. The E6/E7 gRNA guide sequence used were 5’-CAACAGTTACTGCGACGTG-3’ and 5’-TCCGGTTCTGCTTGTCC AGC-3’. SiHa cell lines were transduced twice with first sgRNAE6/E7 lentiviruses, EGFP lentiviruses as the positive control and transfection reagent as the negative control. Puromycin (Merck, Germany) was added on day 4 at a minimal toxic dose to select transduced cells. Then after 3 days of screening, the condition of the cells was observed, and a second transduction performed with Cas9 lentiviruses. Hygromycin at a minimal toxic dose was added after 3 days. The second screening finished after all negative control cells had died. To minimize off-target errors, monoclonal screening was conducted. Single colonies were spread in a 96-well plate and selected by a colony formation assay. The effect of E6/E7 gene editing on cell proliferation was assessed by Trypan Blue viable cell counting over a 7-day time course. The cell apoptosis induction and proliferation inhibition of Ko E6/E7 SiHa cells were markedly induced *in vitro*, which were also cultured in RPIM1640 media (Gibco, US) containing 10% FBS in a 5% CO_2_ incubator at 37°C. Lentiviral transduction efficiency was proven by employing RT-qPCR and western blotting techniques. Total protein from SiHa cells was extracted using RIPA buffer (Solabio, China) and protein expression was determined by western blotting, probed for E6/E7 (Abcam; ab70, ab30731), GAPDH (Yeason, China) using sensitive ECL detection (Yeason, China). Total RNA extraction and the methods of RT-qPCR are described below and the E6 and E7 primers used are listed in **Table S3**.

### Transgene Construction and Production of Transgenic Mice

The diagram illustrating the vector map used to generate transgenic mice was shown in **Figure S1A**. The gene for HPV16 E6/E7 was placed under the control of the K14 promoter using standard methods. The plasmid DNA employed was purified using the method of plasmid midiprep and then used for microinjection into fertilized eggs of C57BL/6×C57BL/6 hybrid mice. The transgenic (Tg) mice were identified by PCR (Transgene PCR primer F1: TCACTCAGCCAACTGCTCGC; Transgene PCR primer R1: GTCGCAGTAACTGTTGCTTGCAG) (**Figure S1B**). Eight founder lines were established by breeding into C57BL/6×C57BL/6 hybrid mice. The line with the highest transgene expression was selected for further analysis. Most experiments were carried out using heterozygous mice that were housed and fed under specific pathogen free environment. In order to identify the phenotype of the cervix tissues, the tissues from the HPV16 E6/E7 transgenic mice were analyzed histologically. The procedures for care and use of animals were approved by the Ethics Committee of the Shenzhen Hospital of Southern Medical University and all applicable conditions according to strict institutional and governmental regulations concerning the ethical use of animals were followed.

### RNA Sequencing

Three copies of SiHa and KoE6/E7 SiHa cell specimens were dissolved in Trizol, and the RNA-seq was determined by the Novo Nordisk Company. The analysis results were evaluated with the aid of the KEGG library. After RNA extraction, the concentration and quality of the total RNA was determined using agarose gel electrophoresis and the RNA stripe was found to be complete, which indicated no degradation. The total RNA purity (OD 260/280) was detected by Nanodrop and the total RNA accurately quantified using Qubit.

#### Complementary DNA Library Preparation and Sequencing

The RNA was first enriched with oligo magnetic beads, then fragmented into small pieces using a fragmentation buffer and first-strand complementary DNA was generated using a primer random template. Second-strand cDNA was synthesized by adding dNTPs and DNA polymerase I and the synthesis of cDNA was purified using AMPure XP beads. Double-stranded cDNA was repaired by adding polyA-sequencing joint. DNA fragments combined with a specifically labeled joint were amplified by PCR and the PCR system was purified using BECKMAN AMPure XP beads. After the establishment of a library, Qubit 2.0 was used for preliminary quantification and then the quality test of the library was conducted using an Agilent 2100 Bioanalyzer machine. The library was quantified by qPCR to ensure its quality and finally HiSeq/MiSeq sequencing for the qualified library was completed. After the quality assessment of the original sequencing data, GO analysis, KEGG enrichment pathway and a protein interaction network were used to analyze the levels and expression of differential proteins.

### Tissue Microarray

Tissue microarrays were obtained from Outdo Biotech Co., Ltd. (Shanghai, People’s Republic of China). IHC studies of PD-L1 were performed on cervical cancer and normal specimens of tissue in a microarray using anti-PD-L1 antibodies (Proteintech). Quantitative analysis of the staining was conducted based on the percentage of positive cells and staining density by three experienced pathologists. For data analysis, staining scores < 2 were defined as low expression and scores > 2 as high expression.

### Immunohistochemistry and HE Staining

After tissues had been collected and fixed, they were typically dehydrated and embedded in melted paraffin wax, and finally cut into thin slices. The slices were affixed to microscope slides at which point the wax was removed with a solvent and the tissue slices were rehydrated. Arrays were dewaxed and then doused with endogenous peroxidase 3% hydrogen peroxide. Followed by blocking using PBS supplemented with 10% goat serum at 30mins, the specific antibody, E6 (Bioss,bs-1719R) and E7 (Bioss, bs-4623R),were incubated at 4°C overnight, and the secondary antibody was incubated at room temperature for 30 min. Cells were washed with PBS, and then incubated with the appropriate secondary antibody for 30 min at room temperature. Then DAB was added for 10 min incubation and cells were finally stained with hematoxylin. Cells were viewed and photographed using a microscope.

### Total RNA Isolation and Quantitative Real-Time PCR

Total RNA was isolated using TRIzol reagent (Invitrogen, US) and cDNA was generated using the Hifair^®^ II 1st Strand cDNA Synthesis SuperMix (Yeason, China). qPCR was performed using a 7500 system (ABI, USA) with Hieff qPCR SYBR Green Master Mix (Yeason, China). The relative expression levels of the target genes were normalized to the expression level of the internal control GAPDH. The data analyses were performed using the 2^−ΔΔCt^ method and the primer sequences are listed in **Supplementary Table 3 (Table S3)**.

### Western Blotting

Cultured cells were lysed with lysis buffer. Equal amounts of protein were run on 10% SDS-PAGE and electrotransferred to polyvinylidene fluoride membranes (Millipore). After blocking with 5% milk in TBST, membranes were incubated with primary antibodies overnight. The following antibodies were used: anti-HPVE6 (1:1000, Arigo), anti-HPVE7(1:1000, Bioss), (anti-CXCL10 (1:1,000, Abcam), anti-CXCR3 (1:1,000, Boster), anti-PDL1 (1:2,000, proteintech), anti-STAT1 (1:1,000, proteintech), anti-pSTAT1 (1:1,000, Abcam), anti-JAK1 (1:2,000, proteintech), anti-GAPDH (1:6,000, Yeasen) and anti-tublin (1:3,000, Yeasen). Membranes were then incubated with the rabbit peroxidase-conjugated secondary antibody (1:10,000, Abclonal). The blots were detected by sensitive chemiluminescence liquid analysis (Yeasen) and Biorad software was used to capture the images.

### siRNA Transfection

The siRNA transfection referred to Ribo Company transfection guidance, and the target sequence of HPVE6 siRNA was GTAGAGAAACCCAGCTGTA. 12 – 16 h prior to transfection, 2 × 10^5^ cells per well were planted on 24-well culture plates. Then a mixture of buffer, siRNA and reagent was prepared and incubated at room temperature for 15 min before being added to cells. After 36 – 48 h post-transfection, the cells were further collected in lysate buffer for western blot analysis.

### Transwell Co-Culture Assay

For transwell co-culture assay, 0.4 μm PC transwell (Corning) was used to do this experiment. The SiHa or Caski cells were planted in upper chamber at a density of 2-3 × 10^5^ cells per well and HFF-1 or Ko E6/E7 SiHa cells were planted in the lower chamber at a density of 2 × 10^5^ cells per well. When all cells were adherent, the cells in the upper chamber and lower chamber were co-cultured together for 48 h though transwell assay. Furthermore, the CXCL10 (AMG487, MCE) (26) and JAK1-STAT1 (Ruxonitilib, MCE) inhibitor (28) were added into the upper chamber at a concentration of 1 μM for 12 h treatment And Recombinant Human CXCL10 (Novoprotein, CX32) were added to the culture media directly at a concentration of 100 ng/ml for 48 h.

### ELISA

Rat IP-10/CXCL10 (Interferon Gamma Induced Protein 10 kDa) ELISA Kit (Elabscience) analysis was performed according to manufacturer’s instructions. For detection of PD-L1 on extracellular vesicles, cell supernatants, 100 µl of extracellular vesicle samples purified from cell culture supernatants, were added to ELISA plates (96-well) (Elabscience) and left to incubate for 1 h and then 100 μL of Biotinylated Detection Ab Diluent against PD-L1 was added and allowed to incubate for 1 h at 37°C. After discarding the liquid in the plate, 100 μL of HRP conjugate was added to each well for a 0.5 h incubation time at 37°C and then 90 μL of substrate was added to each well for 15 min. Plates were stopped with 50 μL of stop solution. The plates were read at 450 nm on a BioTek plate reader and the concentration of ip-10/CXCL10 was in direct proportion to the OD 450 value. The concentration of ip-10/CXCL10 in a sample was calculated against a standard curve.

### Proliferation Assay

For CCK8 assay, the cells were plated at a density of 3,000 cells per well in 96-well plates. After adhesion to wall, 10 µl CCK8 was added into the 100 µl cell culture medium and the cells were incubated for 2 h at 37°C. Then cell viability was determined at a 570 nm wavelength using the CCK-8 assay according to the manufacturer’s instructions (Meilunbio, MA0218-3) (**Figure S3A**).

### Apoptosis Analysis

Apoptosis was assessed by Annexin V staining and flow cytometry analysis. Briefly, 5 × 10^5^ SiHa or KoE6/E7 SiHa cells were harvested, washed in PBS, and then analyzed by Annexin V/propidium iodide staining by flow cytometry according to the manufacturer’s protocol (Annexin V-APC PI kit; Biolegend) (**Figure S3B**).

### Subcutaneous Tumor Formation Studies

The tumor model was established by subcutaneous (s.c.) injection of 5 × 10^5^ SiHa or KoE6/E7 SiHa cells in 120 µl PBS into the flank of nude mice. Tumor volumes were determined from caliper measurements of tumor length (L) and width (W) according to the formula L × W^2/^2. Both tumor size and body weight were measured 3 times each week (**Figure S3C**). At the end of the experiment, tumors were removed and fixed in 4% paraformaldehyde, then the structure of the tissue was determined by means of HE staining and immunohistochemistry (**Figures S3D, E**).

### Purification of Extracellular Vesicles

For exosome purification from cell culture supernatants, cells were cultured in media supplemented with 10% exosome depleted FBS. Supernatants were collected from 48 – 72 h cell cultures and the extracellular vesicles were purified by a standard differential centrifugation protocol (29). In brief, culture supernatants were centrifuged at 2,000 g for 20 min to remove cell debris and dead cells (Beckman Coulter, Allegra X-14R). Microvesicles were pelleted after centrifugation at 10,000g for 30 min (Beckman Coulter, J2-HS) and resuspended in PBS. Supernatants were then centrifuged at 100,000 g for 2 h at 4°C (Beckman Coulter, Optima XPN-100) to collect exosomes and then 100,000 g centrifugation for 2 h was repeated and exosomes washed. The exosomes were dissolved in PBS and stored at -20°C or -80°C.

In addition, approximately 1 ml blood was collected from the heart of mice in order to obtain exosomes from per mouse, using anticoagulation tubes to prevent blood clotting in 17^th^ day after subcutaneous (s.c.) injection. Immediately, the blood was centrifuged at 3,000 rpm for 10 min to remove blood cells, then the upper layer of plasma was gently aspirated, and this procedure was repeated twice. The collected plasma was centrifuged at 10,000 g for 30 min to remove extracellular macrovesicles, then the supernatant was filtered through a 0.22 µm filter and finally centrifuged at 120,000 g for 90 min using an ultra-high speed centrifuge to remove the supernatant. The precipitate was then resuspended with PBS and centrifuged at 120,000 g for 90 min and then supernatant was carefully removed. The precipitate was resuspended with PBS which actually were the exosomes and stored at -80°C.

### Statistical Analysis

C_T_ values were exported to an Excel file to create a table of C_T_ values. This table was then uploaded to the data analysis web portal at http://www.qiagene.com/qiageneglobe. Specimens were assigned to control or test groups. C_T_ values were normalized based on manual selection of reference genes. Statistical evaluations were also conducted using Graphpad Prism (version 7.0) and IBM SPSS software (version 24.0). The Shapiro-Wilk W test was used to determine whether data were normally distributed continuous variables. Levene’s test was used to justify equality of variances. A *t*-test was used to analyze mean values for normally distributed continuous variables and a Mann-Whitney U test to compare mean values for non-normally distributed continuous variables. Correlations between relative mRNA expression and quantitative titer for HPV infection were evaluated by Spearman’s correlation. For all statistical tests, a *P*-value < 0.05 (two-tailed test) was considered to be a statistically significant finding.

## Results

### hrHPV Infection in Different Cervical Lesion Progressions

For 60 specimens constituting 20 each for the normal cervix (NC, A group), HSIL (B group) and CSCC (C group) groups, HPV positive infections were detected in 2, 17 and 20 cases respectively, with high-risk HPV infections in 0, 14 and 17 cases, and low-risk HPV infections in 2, 3 and 3 cases (**Table 1**). The distribution of hrHPV infections in different types of cervical lesions in Xinjiang (n%) were: no hrHPV infections found in 20 patients in the NC group; in the HSIL group, hrHPV infection was primarily HPV16 (40 (%), HPV52 (20%), and HPV31 (15%); the infection rate of HPV16 was significantly higher than in the normal control group (*P* < 0.05) (**Table 2**).

**Table 1:** HPV infection in 60 cases of cervical lesions.

Group	Cases	HPV (+)	HPV (-)	Multiple infections	Single infection	hrHPV infection	Low-risk HPV infection	HPV positive incidence
**NC**	20	2	18	0	2	0	2	10%
**HSIL**	20	17	3	4	13	14	3	85%
**CSCC** ***P*-value**	20	20	0	1	19	17	3	100% < 0.05

**Table 2:** Subtype distribution of high-risk HPV infection in different cervical lesions (n%).

Subtype of hrHPV infection	Normal control(20 cases)	HSIL(20 cases)	CC(20 cases)
16	0	8 (40%)	13 (65%)
18	0	0	8 (40%)
31	0	3 (15%)	1 (5%)
33	0	1 (5%)	0
35	0	0	0
39	0	1 (5%)	1 (5%)
45	0	1 (5%)	1 (5%)
51	0	1 (5%)	0
52	0	4 (20%)	3 (15%)
56	0	0	0
58	0	0	0
59	0	1 (5%)	0
68	0	0	0

### Identification of Upregulated Expression of CXCL10 in HPV+ Cervical Lesion Tissues

**Figure 1:**
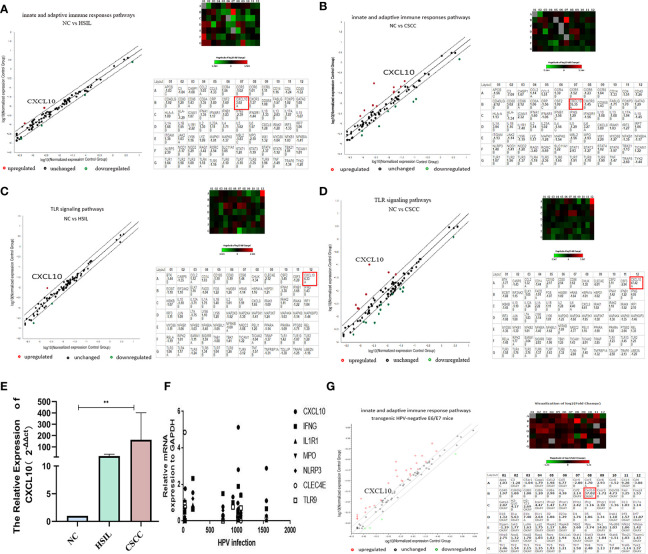
Upregulated expression of CXCL10 in cervical tissues at both the HSIL and CSCC stage; **(A, B)** PCR array analysis of innate and adaptive immune response pathways Heat map showing differentially expressed genes in HSIL or CSCC and Human innate and adaptive immune response pathways gene table (PAHS-052Z) used in RT2 profiler PCR array experiments; **(C, D)** PCR array analysis of the TLR signal transduction pathway (PAHS-018Z); **(E)** The relative expression of CXCL10 in different group by qRT-PCR showed that the mRNA expression of CXCL10 was the highest in CSCC group; **(F)** Correlation analysis between the viral load of HPV infection and the expression of key gene in innate and adaptive immune response pathway including CXCL10; **(G)** Results of PCR array analysis in the innate and adaptive immune response pathways in HPV16 E6/E7 transgenic mice revealed by scatter and cluster diagrams (PAMM-052Z). *P < 0.05, **P < 0.01, ***P<0.001 and ****P < 0.0001, as determined by Student’s t-test.

The results of RT^2^ profiler PCR array confirmed that genes were differentially expressed < 2-fold (downregulation) and > 2-fold (upregulation) in the HSIL (hrHPV+, 14 cases) and CSCC (hrHPV+, 17cases) groups compared to normal control (NC) group, in order to investigate differentially expressed gene of innate and adaptive immune responses related to the microenvironment (receptors, regulatory genes, upstream and downstream effector genes) in HPV+ or HPV- cervical tissues described above (**Table S2A**). After comparison of pairs using the Qiagen tool website, the presented heatmap showed that CXCL10 was significantly overexpressed in CSCC, with a 3.02-fold increase in HSIL *vs* NC (*P* < 0.05), 35-fold in CSCC *vs* NC (*P* < 0.05) and a 12.21 fold increase in CSCC *vs* HSIL (*P* < 0.05) (**Figures 1A, B** and **Table S2C**). Moreover, upregulation of CXCL10 was confirmed in a second assay measuring gene expression of the TLR signaling pathway, which showed a 4.33-fold increase in HSIL *vs* NC and 47.42-fold in CSCC *vs* NC (**Figures 1C, D** and **Table S2B**). And qRT-PCR result substantiated the high expression levels of CXCL10 in CSCC (**Figure 1E**).

The correlations between the relative mRNA expression of differential genes from the PCR array analysis and quantitative titer of HPV infections were evaluated using the Spearman correlation test. Correlation analysis revealed that the viral load of HPV infection only had a statistically significant correlation for the expression of CXCL10 in innate and adaptive immune response signaling pathways (r = 0.662, *P* < 0.05). There was aggregation of gene expression in the concentration area of different HPV infections, which was just at the beginning of the infection stage (0 – 300), and 800 –1,000, 1,500 and so on (**Figure 1F**).

### Upregulated CXCL10 Expression in the Innate and Adaptive Immune Responses in HPV16 E6/E7 Transgenic Mice

To investigate further whether CXCL10 in cervical cancer might be associated with epithelial atypical hyperplasia or carcinogenesis caused by HPV infection (29), HPV16 E6/E7 transgenic mice models, 8 founder lines, were established by breeding into C57BL/6×C57BL/6 hybrid mice. The tissues from the HPV16 E6/E7 transgenic mice were analyzed histologically to characterized consequences of E6 and E7 expression in cervix epithelia, which presented mild epidermal hyperplasia through HE staining (**Figure S1C)** and Immunohistochemistry (**Figure S1D**). Furthermore, cervix tissues from HPV E6/E7 transgenic mice and control groups were also detected with PCR array for innate and adaptive immune response pathways(QIAGEN, Cat. no. PAMM-052Z). Surprisingly, compared to normal mice, the cervical tissues from HPV E6/E7 transgenic mice expressed a 37.23-fold increase in CXCL10 (*P* < 0.05) (**Figure 1G**). In sum, our findings demonstrated significantly increased levels of CXCL10 by HPV-transformed cervical epithelium (**Table S2**). Notably, in the innate immune response pathway, IRF7 and MX1 were greatly increased, which act as IFN regulatory factors and as anti-myxovirus proteins. Others have shown that it was pivotal for the regulation of the IFN responses to evoke antiviral effect in innate immunity (30, 31).

### Elevated Secretion of CXCL10 in HPV+ CSCC Cells

**Figure 2:**
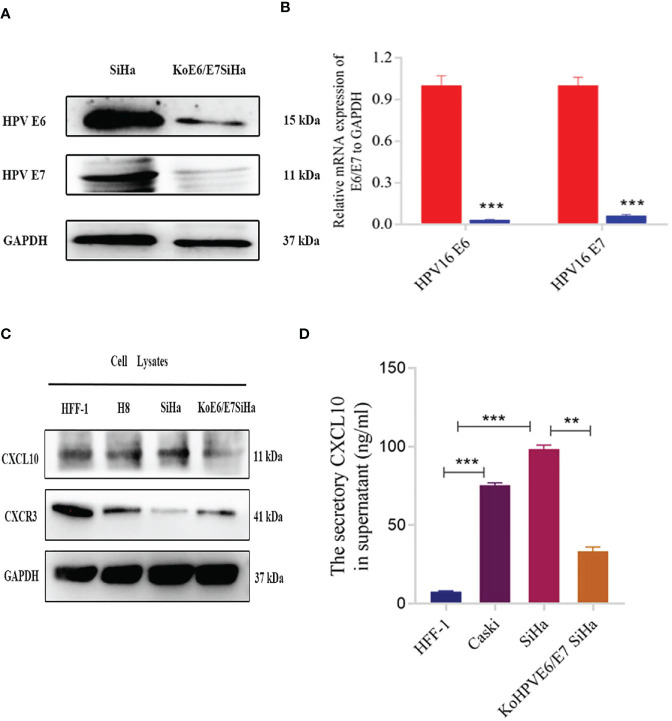
Expression of CXCL10 in different cells revealed by western blotting and Elisa. **(A, B)** The results of western blotting and qPCR verified that the KoE6/E7SiHa cell line was established successfully by CRISPR/Cas9 gene editing; **(C)** The expression of CXCL10 was tested through western blotting in different cell lines including HFF-1, H8, SiHa and KoE6/E7 SiHa; **(D)** The result of Elisa displayed increased secretion of CXCL10 in cervical cancer cells (SiHa and Caski), while was reverse in koE6/E7SiHa cell. *P < 0.05, **P < 0.01, ***P<0.001 and ****P < 0.0001, as determined by Student’s t-test.

To evaluate whether HPV infection was able to modulate the secretion of CXCL10 in HPV+ cells, KoE6/E7SiHa, a HPV E6/E7 knockout SiHa cell line was established through CRISPR/Cas9 gene editing and the knockout efficiency of the E6 and E7 gene by qPCR was 96.7% and 93.6%, respectively (**Figures 2A, B**). Actually, KoE6/E7 SiHa cells grew at a significantly slower rate than SiHa cells *in vitro* through CCK-8 assay and Annexin V staining apoptosis assay, suggesting that the cell apoptosis induction and proliferation inhibition were markedly induced by the knockout of E6 and E7 (**Figures S3A, B**). CXCL10 expression was determined by western blotting of HFF-1 (HPV- human epithelial foreskin fibroblasts cells), H8 (HPV+, cervical immortalized cells), SiHa (HPV+, cervical cancer cells), KoE6/E7 SiHa (HPV-E6E7 knockout cells) and the results showed that the expression of CXCL10 was greater in HPV+ compared to HPV-cell lysates (**Figure 2C**). Next, to verify further whether HPV infection was responsible for higher CXCL10 secretion in HPV+ cells, the supernatants of cells including HFF-1, SiHa, Caski as well as KoE6/E7 SiHa cells were collected to detect the expression of CXCL10 using ELISA. The results demonstrated that the secretion of CXCL10 increased in HPV+ cells and decreased in the HPV-group (**Figure 2D**). In contrast, CXCR3, as the receptor of CXCL10, was expressed more abundantly in HPV- cells (**Figure 2C**). Surprisingly, the results indicated that CXCL10 was mainly expressed and secreted by HPV+ cells while CXCR3 was preponderant in HPV- cells (**Figures 2C, D**). Thus, we hypothesized that HPV+ cells might have a remarkable influence on HPV- cells through complex CXCL10-CXCR3 interactions.

### HPV-Mediated Expression of CXCL10 and Signaling *via* CXCR3 Induces PD-L1 Expression

**Figure 3:**
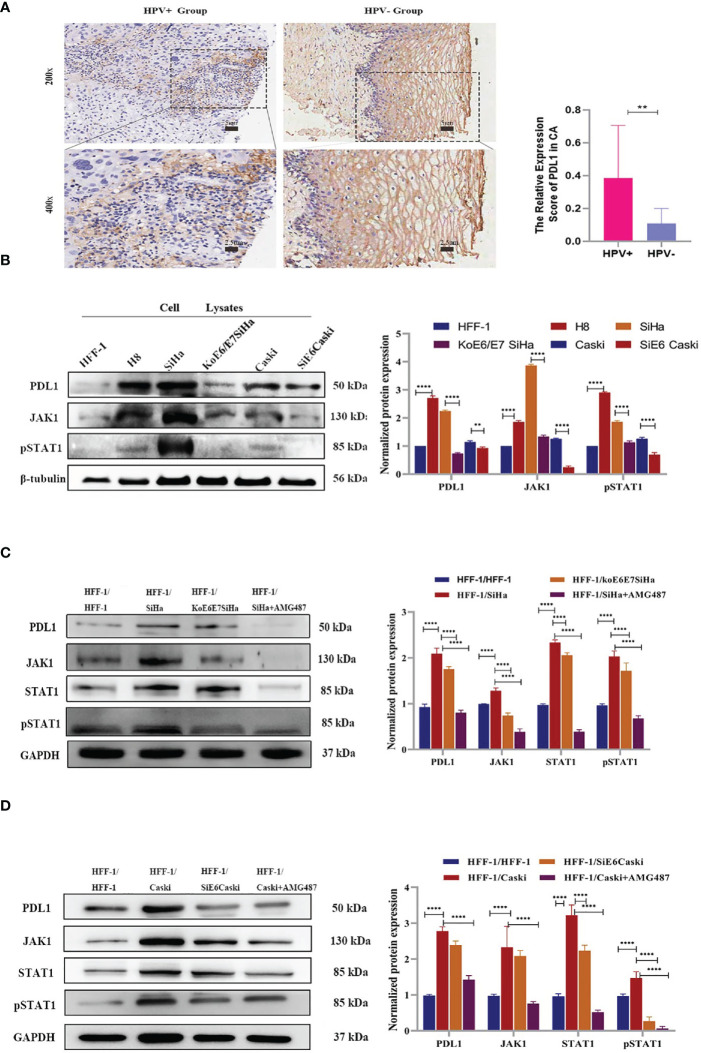
Through transwell co-culture with HPV+ cells (SiHa and Caski), PD-L1 expression in HFF-1 cells were induced by CXCL10-CXCR3 interaction, while were decreased after co-culture with the HPV+ cells which E6 and E7 has been knocked down or treatment with AMG487. **(A)** Immunohistochemistry revealed that the expression of PD-L1 had a strong relationship with HPV infection in cervix tissues, which showed the higher expression of PD-L1 in CSCC (HPV+ Group) than that of a normal cervical epithelium (HPV- Group); **(B)** The expression of PD-L1, JAK1 and pSTAT1 in different cells was tested by western blotting. The band intensities were calculated using the ImageJ software. GAPDH was used as an internal control for the total protein measurement. The ratio of the target gene to GAPDH was used to conduct the statistical analysis. **(C, D)**. Compared to the co-culture with HFF-1 cells, the expression levels of PD-L1, JAK1 and pSTAT1 in HFF-1 cells were all upregulated following co-culture with HPV+ cells (SiHa, Caski) while the upregulation of JAK1 and pSTAT1 were diminished after co-culture with koE6/E7Siha and SiE6Caski cells or treatment with AMG487 using the 0.4 µm polycarbonate membrane transwell assay, in which cells could not pass through. *P < 0.05, **P < 0.01, ***P<0.001 and ****P < 0.0001, as determined by Student’s t-test.

It has been reported that HPV+ tumors tend to have an increased tumor-associated immune cell infiltrate relative to HPV- tumors (32, 33). However, the mechanisms responsible for these differences have not been fully elucidated. In the present study, a total of 25 specimens of cervical squamous cell carcinoma (CSCC) were collected from 14 HPV+ and 11 HPV- patients (**Table S1**). Immunohistochemistry revealed that in CSCC lesions, the expression of PD-L1 had a strong relationship with HPV infection (**Figure 3A**). *In vitro*, the expression of PD-L1 showed that upregulated expression of PD-L1 tended to occur mainly in HPV+ cells (**Figure 3B**).

Actually, this study showed a significantly increased production of multiple chemokines including CXCL10 in HPV+ specimens as well as in transgenic mice. It has been reported that CXCL9/10/11 is a regulator of PD-L1 expression in gastric cancer (14, 28). To explore whether the secretion of CXCL10 was responsible for the expression of PD-L1, a transwell co-culture assay was performed for 48 h using HFF-1 and KoE6/E7 SiHa cells. As shown in **Figure 3B**, after co-culture with HPV+ cells (SiHa, Caski), PD-L1 expression in HFF-1 was upregulated (**Figures 3C, D**). However, when the SiHa or Caski cells cultured in the chamber of a transwell were treated with 1 μM AMG487, which prevents CXCL10-CXCR3 binding, the upregulation of PD-L1 was suppressed (**Figures 3C, D**). To confirm further the specific effect of CXCL10, recombinant human CXCL10 at a concentration of 100 ng/ml was directly added to the culture of HFF-1 and KoE6/E7 SiHa cells for 48 h, respectively and then treatment results after the application of 1 μM AMG487 were subsequently processed. The results showed that PD-L1 was upregulated by CXCL10 and downregulated after AMG487 treatment as expected, suggesting that HPV infection can induce PD-L1 expression by HPV+ cervical cancer through CXCL10-CXCR3 interactions.

### CXCL10 Secreted by HPV+ CSCC Cells Enhanced PD-L1 Expression by CXCL10-CXCR3 Activating the JAK1-STAT Pathway

**Figure 4:**
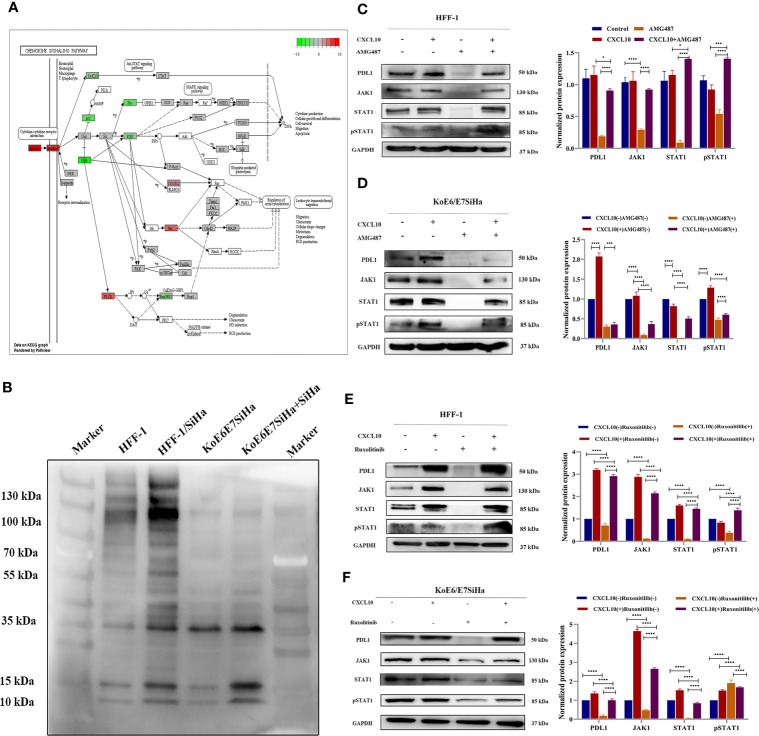
CXCL10-CXCR3 upregulated PD-L1 expression by activating the JAK1-STAT pathway in HPV+ cervical cancer cells and tissues. **(A)** Map of chemokine signaling pathways generated by KEGG pathway analysis in DAVID by RNA-seq from Aksomics company; **(B)** The results of pan-phosphorylation western blot detection on the cells including HFF-1, HFF-1 co-cultured with SiHa, KoE6/E7 SiHa, and KoE6E7SiHa co-cultured with SiHa cells. **(C–F)** The expression of PD-L1, JAK1 and STAT1 in HFF-1 or KoE6/E7 SiHa cells after treatment with recombinant human CXCL10, AMG487 or ruxonitilib, to further verify the HPV E6/E7 can induce upregulated expression of PD-L1 in CSCC through CXCL10 binding to CXCR3 which leads to JAK-STAT pathway activation. *P < 0.05, **P < 0.01, ***P<0.001 and ****P < 0.0001, as determined by Student’s t-test.

According to the map of the KEGG pathway in the DAVID database, the JAK-STAT pathway acts as a downstream pathway for the chemokine-chemokine R downstream pathway (CXCL10-CXCR3) (**Figure 4A**). The difference of pan-phosphorylation among the cell lines including HFF-1, HFF-1 co-cultured with SiHa, KoE6/E7 SiHa, and KoE6E7SiHa co-cultured with SiHa cells revealed that the phosphorylated proteins of the cells co-cultured with SiHa were significantly increased, which indicated that some proteins of the HPV- cells could be phosphorylated and activated by the key protein of HPV+ cells (**Figure 4B**) (34). Interestingly, the PCR array results showed that STAT1 was precisely overexpressed in CSCC *vs* the NC or HSIL groups (*P < 0.05*). Therefore, we hypothesized that CXCL10 may regulate PD-L1 expression through the JAK-STAT pathway (**Table S2**).

In order to verify the JAK-STAT pathway, HFF-1 and KoE6/E7 SiHa cells were treated with recombinant human CXCL10. Notably, targeting JAK proteins or CXCR3 met with success in decreasing the expression of JAK1 and pSTAT1 after treatment with AMG487 (**Figures 4C, D**) and ruxonitilib (**Figures 4E, F**). Therefore, our findings demonstrated a correlation between suppression of STAT1 activation after JAK1 inhibition, evidenced by STAT phosphorylation, and downregulation of PD-L1. Moreover, the effect of the inhibitor on cells was reversed by recombinant human CXCL10, suggesting that CXCL10-CXCR3 interaction play an important role in PD-L1 upregulation by activating the JAK1-STAT1 pathway in HPV- cells.

### PD-L1 Is Specifically Secreted and Transported by Exosomes in HPV+ CSCC

**Figure 5:**
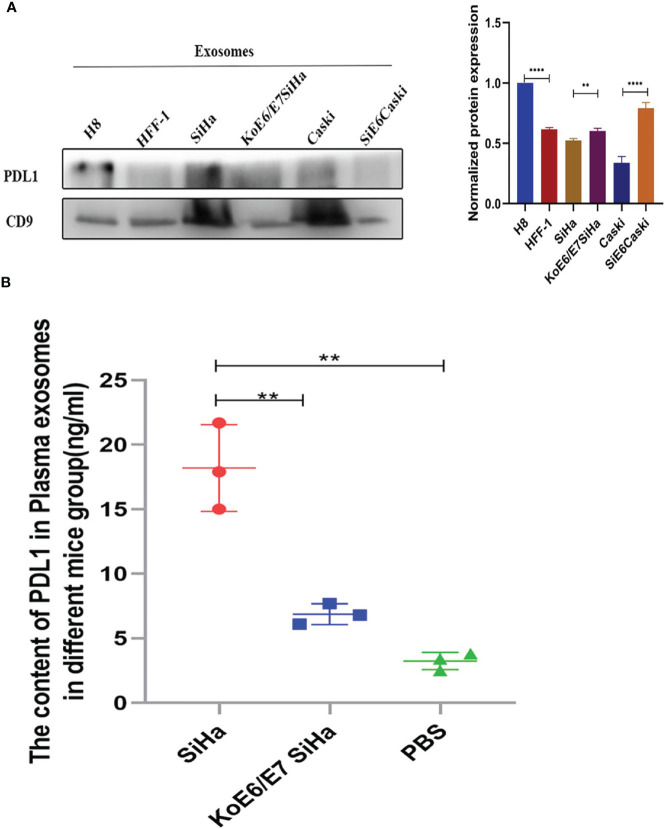
Expression levels of PD-L1 in the NC, SiHa and KoE6/E7 SiHa groups. **(A)** Western blotting showed that PD-L1 was present in cell exosomes; **(B)** ELISA revealed that the expression levels of exosomal PD-L1 was higher in the SiHa subcutaneous tumor group than that in KoE6/E7 SiHa, which were obtained by approximately 1 ml blood collected from the heart of mice from per mouse. *P < 0.05, **P < 0.01, ***P < 0.001 and ****P < 0.0001, as determined by Student’s t-test.

Interestingly, PD-L1 could not be directly detected in the cell supernatant, mentioned above, by ELISA. Our evidence has shown that PD-L1 existed in cell exosomes (**Figure 5A**) (**Figure S2**). Next, a tumor model was established by subcutaneous injection of 5 × 10^5^ SiHa or KoE6/E7 SiHa cells in 120 μl of PBS into the flanks of nude mice (**Figures S3C, D**). After collecting the exosomes, the expression level of PD-L1 was higher in the SiHa group, followed by the KoE6/E7SiHa group, a finding which implied that PD-L1 could be packaged into exosomes in CSCC (**Figure 5B**).

## Discussion

Persistent HPV infection is involved in the pathogenesis of cervical cancer and is strongly associated with its prognosis (35). The oncogenic protein hrHPV E6/E7 may disrupt the regulation of gene expression in host cells that leads to abnormal cell proliferation by affecting the mechanisms involved in the control of the cell cycle and antigen presentation, rendering the body in a state of unresponsiveness (5, 35). Thus, tumor cells will evade attack by the immune system of the host and cancer immune surveillance, which is known as tumor immune escape, leading to the induction of tumorigenesis and progression (36). A number of investigators have proposed the cancer immunoediting hypothesis, namely that during tumor growth the microenvironment simultaneously aids tumor escape from immune surveillance (37). Several research groups have investigated whether HPV infection could affect PD-L1 expression in cervical cancer and reported that HPV positivity was positively correlated with increased PD-L1 expression (38, 39). This evidence indicates a potential role for PD-1/PD-L1 interactions in creating an “immune-privileged” site for initial HPV infection and subsequent adaptive immune resistance (40). Therefore, it is important to elucidate the regulatory mechanism between HPV infection and PD-L1. Our study showed that PD-L1 expression was enhanced by HPV infection *via* the chemokine subfamily CXCL10-CXCR3 axis in a JAK and STAT-dependent manner in CSCC cells, suggesting that chemokines may induce PD-L1 expression through JAK-STAT pathways.

The CXC chemokine ligand 10, also known as IFN-γ-inducible protein 10 (IP-10), is an IFN-induced protein having the ability to trigger lymphocyte chemotaxis (41). Previous studies have shown that CXCL10 has critical biological functions, such as selective expression of CXCR3 on Th1 cells and the regulation of immune cell migration, differentiation and activation (38, 41). CXCL10 has long been known to exert anti-malignancy functions by influencing the tumor microenvironment. Recently, some researchers have taken a different approach and investigated the direct effects of CXCL10 on tumor-promoting functions in colorectal carcinoma cells (42). The data supported the potential for CXCL10/CXCR3 co-expression as a predictor of metastatic recurrence (17). However, in the present study, the expression levels of CXCL10 in cervical lesions both in the HPV+ HSIL and CSCC groups as well as in HPVE6/E7 transgenic mice were significantly increased in the innate and adaptive signaling pathways. In addition, the secretion of CXCL10 was more apparent in HPV+ cervical cancer cells compared to non-infected and immortal epithelial cells. After dealing with SiHa cells by CRISPR/Cas9 gene editing, the E6/E7 knockout resulted in a major decrease of CXCL10 in KoE6/E7 SiHa, indicating a crucial function of HPVE6/E7 in the regulation of CXCL10 in human cervical cells as well.

Interestingly, our study found that PD-L1 was overexpressed in CSCC cells and CSCC tissues infected with HPV, especially in the exosome of CSCC cells as well as in the plasma of transplanted tumor mice. This novel HPV E6/E7-mediated mechanism of action may have developed to strengthen the secretion of the chemokine CXCL10 in the innate and adaptive immune response pathways. This allows the CXCL10-CXCR3 axis to upregulate PD-L1 expression by activating JAK and STAT pathways in fibroblasts to facilitate viral latency and avoidance of immune system viral attack. The finding is of significant interest to the field, challenging the paradigm that the effects of CXCL10 are anti-tumor immune responses (43) through immunosuppressive mechanisms, including upregulated expression of PD-L1.

## Conclusions

In summary, our results from data analysis and *in vitro* and *in vivo* experiments have suggested a regulatory mechanism of HPV infection-related upregulation of PD-L1, which is an important factor for immune evasion in HPV+ CSCC cells. CXCL10-CXCR3 may well regulate PD-L1 expression through JAK and STAT signaling pathways in fibroblasts cells. Checkpoint blockers such as PD-L1 offer novel immunotherapy options for cancer patients. Based on our findings, CXCL10-CXCR3 could be a potential target for CSCC therapy but further studies will be required to confirm this conjecture.

## Data Availability Statement

The raw data supporting the conclusions of this article will be made available by the authors, without undue reservation.

## Ethics Statement

The animal study was reviewed and approved by Shenzhen Hospital, Southern Medical University.

## Author Contributions

Conceived and designed the experiments: YH, XG, XL. Performed the experiments: XG, YX, XNC, MY. Analyzed the data: XG, XY and HH. Contributed reagents/materials/analysis tools: YH, XG, AH, JLY. Wrote the paper: XNC, XG, YX. Review and Editing: XNC, HH. All authors contributed to the article and approved the submitted version.

## Funding

This study was supported by the National Natural Science Foundation of China (No.81972423); the project of free exploration from Shenzhen Technology Innovation Committee (JCYJ20170307144103633, JCYJ20190814111801681, JCYJ20190814110207603); the clinical research start-up plan of Southern Medical University (Grant no. LC2016YM018); a grant from the Shenzhen Key Laboratory of Viral Oncology (ZDSYS201707311140430) and a grant from the Sanming Medical Project (SM201702). The funding parties had no influence on the study design, data collection, analysis or interpretation of the results.

## Conflict of Interest

The authors declare that the research was conducted in the absence of any commercial or financial relationships that could be construed as a potential conflict of interest.

## Publisher’s Note

All claims expressed in this article are solely those of the authors and do not necessarily represent those of their affiliated organizations, or those of the publisher, the editors and the reviewers. Any product that may be evaluated in this article, or claim that may be made by its manufacturer, is not guaranteed or endorsed by the publisher.

## Supplementary Material

Supplementary Figure 1 The establishment of HPV E6e7 transgenic mice; **(A)** Diagram illustrating the vector map used to generate transgenic mice; **(B)** Verification of the HPV16 E6/E7 transgenic mice by PCR array; **(C, D)** The cervix tissues from the normal mice(NC) and the HPV16 E6/E7 transgenic mice(Tg) were analyzed histologically through HE staining and Immunohistochemistry, and the result of the HPV16 E6/E7 transgenic mice presented Basal cells with large, deeply stained nucleus gently.

Supplementary Figure 2 Identification of the exosomes by electron microscopy and markers; **(A) **The representative transmission electron microscopy image displaying the morphology and size of exosomes; Scale bars, 200 nm; **(B)** Result displaying the CD63 and CD9 (exosomal marker) immunoblot in H8 and SiHa cell lines.

Supplementary Figure 3 The results of tumor model by subcutaneous injection of 5 × 10^5^ SiHa or KoE6/E7 SiHa cells into nude mice. **(A, B)** The proliferation and apoptosis of SiHa and KoE6/E7 SiHa cells were detected by CCK-8 assay and Annexin V staining apoptosis assay; **(C)** The volume of tumor was determined from caliper measurements of tumor length (L) and width (W) according to the formula L × W^2^/2; **(D)** The histology of tumors were detected by HE staining and **(E)** The expression of HPV oncogenes E6 and E7 was determined by immunohistochemistry, which further presented the higher expression of E6 and E7 protein in SiHa group than that in koE6/E7SiHa group.

## Abbreviations

HPV, human papilloma virus; PD-L1, Programmed death-ligand 1; CSCC, Cervical squamous-cell carcinoma; CIN, cervical intraepithelial neoplasia; HSIL, high grade squamous intraepithelial lesion; CXCL10, CXC motif chemokine 10; CXCR3, CXC chemokine receptor-3; Th cells, T helper cells; CTLs, cytotoxic T lymphocytes; NKs, natural killer cells; PC, pancreatic cancer; HCC, hepatocellular carcinoma; PTC, papillary thyroid carcinoma.
