# Supplementary figures and images for: CXCL10 Produced by HPV-Positive Cervical Cancer Cells Stimulates Exosomal PDL1 Expression by Fibroblasts via CXCR3 and JAK-STAT Pathways
Source: Front Oncol. 2021 Aug 6;11:629350. doi: 10.3389/fonc.2021.629350 (PMC8377428; doi:10.3389/fonc.2021.629350)

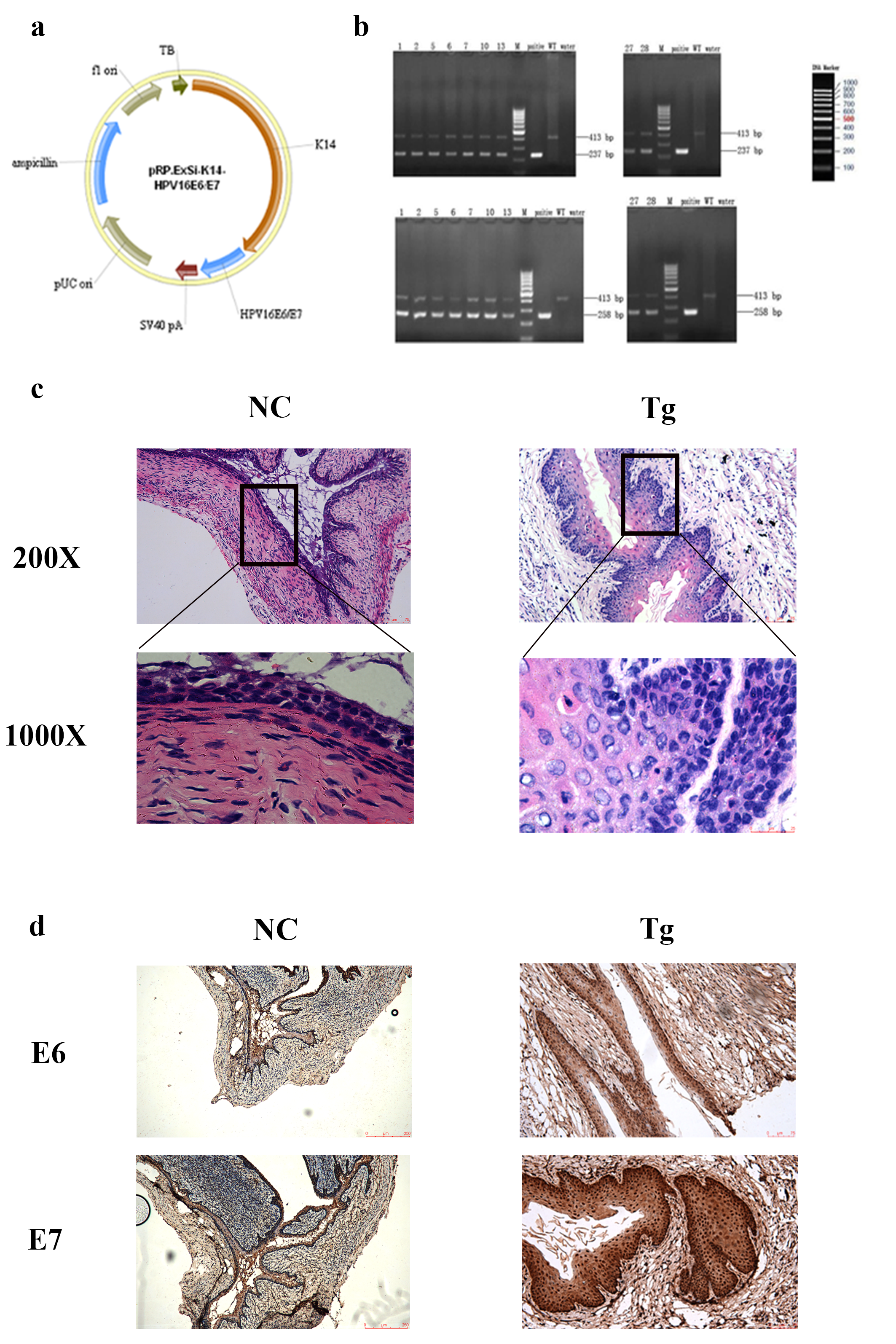

Supplement: Supplementary Figure 1 — The establishment of HPV E6e7 transgenic mice; (A) Diagram illustrating the vector map used to generate transgenic mice; (B) Verification of the HPV16 E6/E7 transgenic mice by PCR array; (C, D) The cervix tissues from the normal mice(NC) and the HPV16 E6/E7 transgenic mice(Tg) were analyzed histologically through HE staining and Immunohistochemistry, and the result of the HPV16 E6/E7 transgenic mice presented Basal cells with large, deeply stained nucleus gently. [file Image_1.tif]

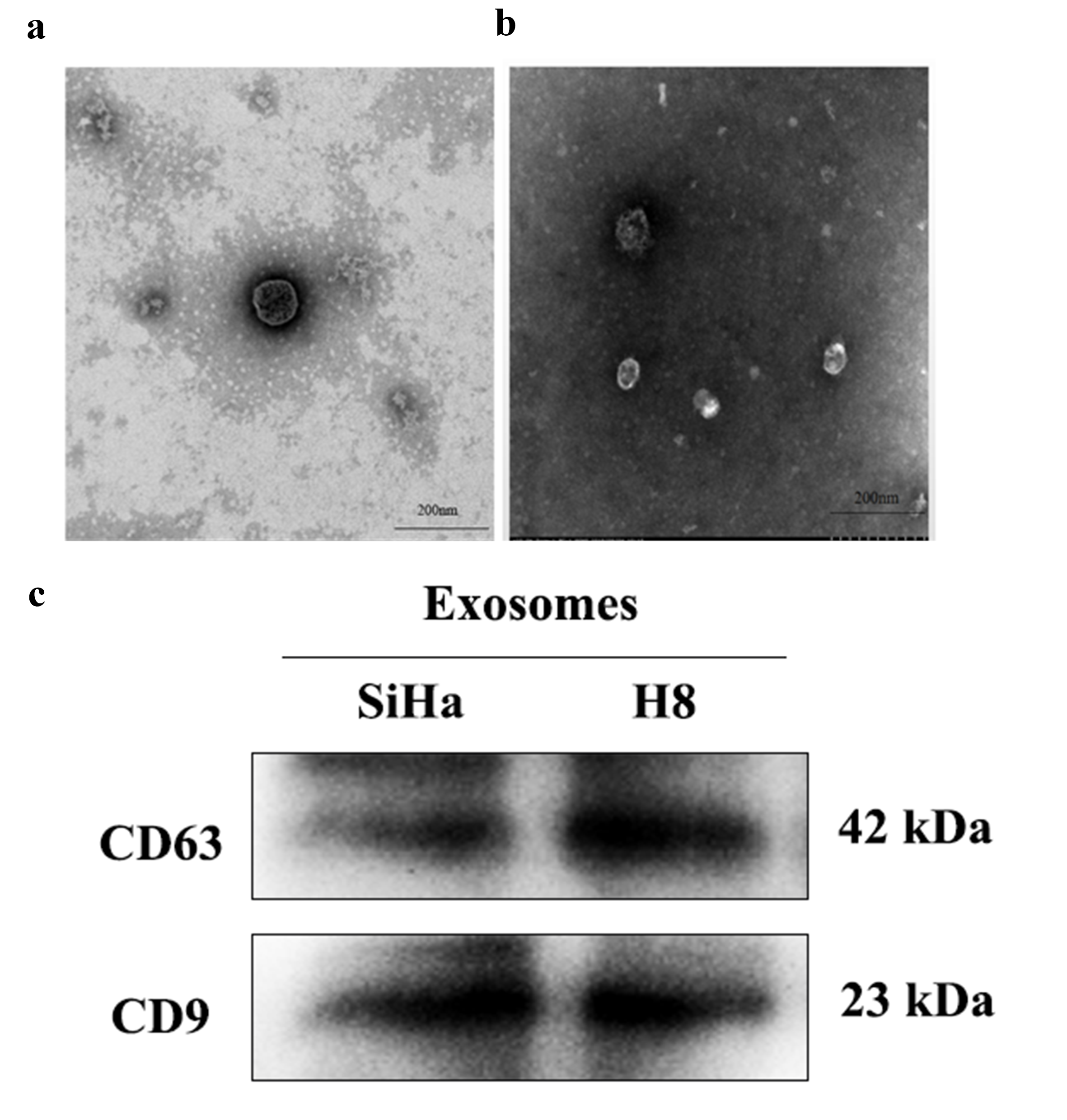

Supplement: Supplementary Figure 2 — Identification of the exosomes by electron microscopy and markers; (A) The representative transmission electron microscopy image displaying the morphology and size of exosomes; Scale bars, 200 nm; (B) Result displaying the CD63 and CD9 (exosomal marker) immunoblot in H8 and SiHa cell lines. [file Image_2.tif]

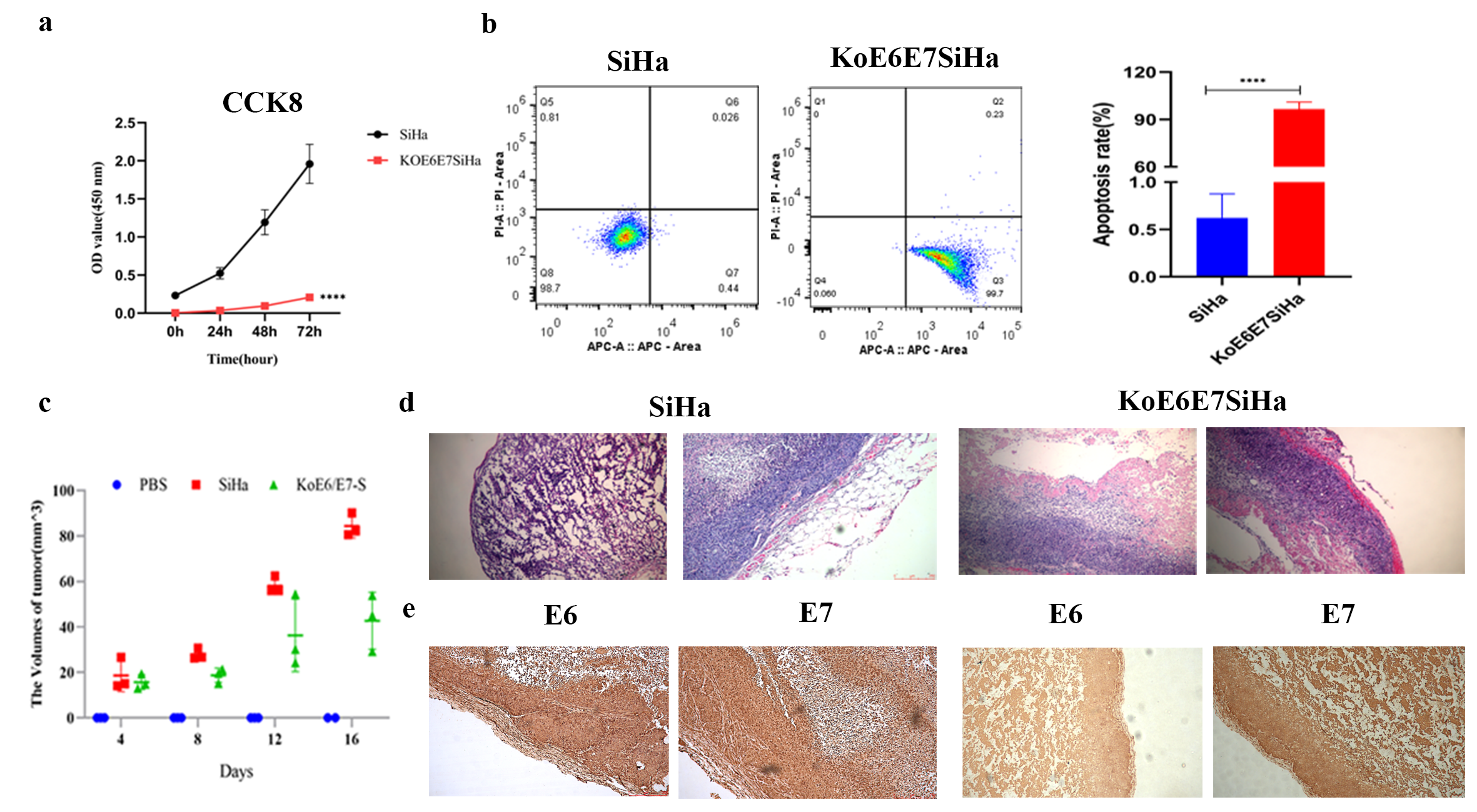

Supplement: Supplementary Figure 3 — The results of tumor model by subcutaneous injection of 5 × 10^5^ SiHa or KoE6/E7 SiHa cells into nude mice. (A, B) The proliferation and apoptosis of SiHa and KoE6/E7 SiHa cells were detected by CCK-8 assay and Annexin V staining apoptosis assay; (C) The volume of tumor was determined from caliper measurements of tumor length (L) and width (W) according to the formula L × W2/2; (D) The histology of tumors were detected by HE staining and (E) The expression of HPV oncogenes E6 and E7 was determined by immunohistochemistry, which further presented the higher expression of E6 and E7 protein in SiHa group than that in koE6/E7SiHa group. [file Image_3.tif]
