# Supplementary material for: CXCL10 Produced by HPV-Positive Cervical Cancer Cells Stimulates Exosomal PDL1 Expression by Fibroblasts via CXCR3 and JAK-STAT Pathways
Source: Front Oncol. 2021 Aug 6;11:629350. doi: 10.3389/fonc.2021.629350 (PMC8377428; doi:10.3389/fonc.2021.629350)
Supplement: Supplementary file 4 [file Table_1.docx]

**Supplementary Table S1(a)**

25 samples of cervical squamous cell carcinoma(CSCC)

| Number | Age(year) | Event | Survival time(month) | [Pathological type](http://dict.cnki.net/dict_result.aspx?searchword=%e7%97%85%e7%90%86%e5%88%86%e5%9e%8b&tjType=sentence&style=&t=pathological+type) | Pathological grade | HPV | HUteS168Su01（PDL1） | HUteS168Su01（PDL1） |
| --- | --- | --- | --- | --- | --- | --- | --- | --- |
| 1 | 42 | alive | 84 | [squamous carcinoma](http://dict.cnki.net/dict_result.aspx?searchword=%e9%b3%9e%e7%99%8c&tjType=sentence&style=&t=squamous+carcinoma) | Ⅲ | 16 | 0.5 | 30% |
| 2 | 38 | alive | 81 | [squamous carcinoma](http://dict.cnki.net/dict_result.aspx?searchword=%e9%b3%9e%e7%99%8c&tjType=sentence&style=&t=squamous+carcinoma) | Ⅱ | 18 | 1.5 | 90% |
| 3 | 47 | death | 48 | [squamous carcinoma](http://dict.cnki.net/dict_result.aspx?searchword=%e9%b3%9e%e7%99%8c&tjType=sentence&style=&t=squamous+carcinoma) | Ⅰ-Ⅱ | 18、16 | 0.5 | 20% |
| 4 | 35 | alive | 77 | [squamous carcinoma](http://dict.cnki.net/dict_result.aspx?searchword=%e9%b3%9e%e7%99%8c&tjType=sentence&style=&t=squamous+carcinoma) | Ⅲ | 16 | 0.5 | 30% |
| 5 | 39 | alive | 76 | [squamous carcinoma](http://dict.cnki.net/dict_result.aspx?searchword=%e9%b3%9e%e7%99%8c&tjType=sentence&style=&t=squamous+carcinoma) | Ⅱ-Ⅲ | （-） | 0.5 | 10% |
| 6 | 55 | death | 45 | [squamous carcinoma](http://dict.cnki.net/dict_result.aspx?searchword=%e9%b3%9e%e7%99%8c&tjType=sentence&style=&t=squamous+carcinoma) | Ⅲ | 16 | 1 | 60% |
| 7 | 29 | alive | 74 | [squamous carcinoma](http://dict.cnki.net/dict_result.aspx?searchword=%e9%b3%9e%e7%99%8c&tjType=sentence&style=&t=squamous+carcinoma) | Ⅲ | 16 | 1.5 | 30% |
| 8 | 48 | death | 43 | [squamous carcinoma](http://dict.cnki.net/dict_result.aspx?searchword=%e9%b3%9e%e7%99%8c&tjType=sentence&style=&t=squamous+carcinoma) | Ⅲ | （-） | 0 | 0% |
| 9 | 53 | alive | 84 | [squamous carcinoma](http://dict.cnki.net/dict_result.aspx?searchword=%e9%b3%9e%e7%99%8c&tjType=sentence&style=&t=squamous+carcinoma) | Ⅰ-Ⅱ | （-） | 0.5 | 10% |
| 10 | 46 | alive | 84 | [squamous carcinoma](http://dict.cnki.net/dict_result.aspx?searchword=%e9%b3%9e%e7%99%8c&tjType=sentence&style=&t=squamous+carcinoma) | Ⅲ | （-） | 1 | 20% |
| 11 | 41 | death | 64 | [squamous carcinoma](http://dict.cnki.net/dict_result.aspx?searchword=%e9%b3%9e%e7%99%8c&tjType=sentence&style=&t=squamous+carcinoma) | Ⅲ | （-） | 1 | 20% |
| 12 | 47 | death | 56 | [squamous carcinoma](http://dict.cnki.net/dict_result.aspx?searchword=%e9%b3%9e%e7%99%8c&tjType=sentence&style=&t=squamous+carcinoma) | Ⅱ-Ⅲ | （-） | 0 | 0% |
| 13 | 56 | alive | 76 | [squamous carcinoma](http://dict.cnki.net/dict_result.aspx?searchword=%e9%b3%9e%e7%99%8c&tjType=sentence&style=&t=squamous+carcinoma) | Ⅲ | 16 | 1 | 20% |
| 14 | 60 | death | 37 | [squamous carcinoma](http://dict.cnki.net/dict_result.aspx?searchword=%e9%b3%9e%e7%99%8c&tjType=sentence&style=&t=squamous+carcinoma) | Ⅲ | 18 | 0.5 | 35% |
| 15 | 49 | death | 24 | [squamous carcinoma](http://dict.cnki.net/dict_result.aspx?searchword=%e9%b3%9e%e7%99%8c&tjType=sentence&style=&t=squamous+carcinoma) | Ⅲ | （-） | 0.5 | 50% |
| 16 | 46 | alive | 72 | [squamous carcinoma](http://dict.cnki.net/dict_result.aspx?searchword=%e9%b3%9e%e7%99%8c&tjType=sentence&style=&t=squamous+carcinoma) | Ⅲ | 18 | 1 | 40% |
| 17 | 57 | death | 42 | [squamous carcinoma](http://dict.cnki.net/dict_result.aspx?searchword=%e9%b3%9e%e7%99%8c&tjType=sentence&style=&t=squamous+carcinoma) | Ⅱ-Ⅲ | 18 | 0.5 | 20% |
| 18 | 42 | alive | 71 | [squamous carcinoma](http://dict.cnki.net/dict_result.aspx?searchword=%e9%b3%9e%e7%99%8c&tjType=sentence&style=&t=squamous+carcinoma) | Ⅲ | （-） | 0.5 | 30% |
| 19 | 61 | death | 39 | [squamous carcinoma](http://dict.cnki.net/dict_result.aspx?searchword=%e9%b3%9e%e7%99%8c&tjType=sentence&style=&t=squamous+carcinoma) | Ⅲ | 18 | 1 | 60% |
| 20 | 67 | death | 29 | [squamous carcinoma](http://dict.cnki.net/dict_result.aspx?searchword=%e9%b3%9e%e7%99%8c&tjType=sentence&style=&t=squamous+carcinoma) | Ⅲ | 16 | 1 | 30% |
| 21 | 43 | alive | 68 | [squamous carcinoma](http://dict.cnki.net/dict_result.aspx?searchword=%e9%b3%9e%e7%99%8c&tjType=sentence&style=&t=squamous+carcinoma) | Ⅱ | 16 | 0.5 | 60% |
| 22 | 52 | alive | 68 | [squamous carcinoma](http://dict.cnki.net/dict_result.aspx?searchword=%e9%b3%9e%e7%99%8c&tjType=sentence&style=&t=squamous+carcinoma) | Ⅲ | （-） | 1 | 15% |
| 23 | 47 | alive | 66 | [squamous carcinoma](http://dict.cnki.net/dict_result.aspx?searchword=%e9%b3%9e%e7%99%8c&tjType=sentence&style=&t=squamous+carcinoma) | Ⅱ-Ⅲ | （-） | 2 | 1% |
| 24 | 63 | death | 37 | [squamous carcinoma](http://dict.cnki.net/dict_result.aspx?searchword=%e9%b3%9e%e7%99%8c&tjType=sentence&style=&t=squamous+carcinoma) | Ⅲ | 16 | 1 | 30% |
| 25 | 47 | alive | 65 | [squamous carcinoma](http://dict.cnki.net/dict_result.aspx?searchword=%e9%b3%9e%e7%99%8c&tjType=sentence&style=&t=squamous+carcinoma) | Ⅲ | （-） | 0.5 | 10% |

**Supplementary Table S1(b)**

HPV subtypes of 20 CSCC cases

| **CSCC cases** | **Subtype of**  **HPV infection** |  | **CSCC cases** | **Subtype of**  **HPV infection** |
| --- | --- | --- | --- | --- |
| 1 | 16,31 |  | 11 | 16,18 |
| 2 | 16,18 |  | 12 | 16 |
| 3 | 42 |  | 13 | 18,52 |
| 4 | - |  | 14 | 52 |
| 5 | 18 |  | 15 | 16,39 |
| 6 | 16 |  | 16 | 16 |
| 7 | 16,18 |  | 17 | 16,45 |
| 8 | 40 |  | 18 | 16,18 |
| 9 | 16 |  | 19 | 16,18,52 |
| 10 | 16 |  | 20 | 18 |

**Supplementary Table S2**

Functional over-expressed gene groupings in PCR Array Profiling of innate and adaptive immune responses pathway (a,c) and Toll-Like Receptor Signaling Pathway(b,d) in CSCC tissues .

**Innate and adaptive immune responses pathway (a)**

| **Innate Immunity:**  Pattern Recognition Receptors: DDX58 (RIG-I), NLRP3, NOD1 (CARD4), NOD2, TLR1, TLR2, TLR3, TLR4, TLR5, TLR6, TLR7, TLR8, TLR9.  Cytokines: CCL2 (MCP-1), CCL5 (RANTES), CSF2 (GM-CSF), **CXCL10,** IFNA1, IFNB1, IL18, IL1A, IL1B, IL2, IL8, TNF.  Other Genes: APCS, C3, CASP1 (ICE), CD14, CD4, CD40 (TNFRSF5), CD40LG (TNFSF5), CD8A, CRP, HLA-A, HLA-E, IL1R1, IRAK1, IRF3, IRF7, ITGAM, LY96 (MD-2), LYZ, MAPK1 (ERK2), MAPK8 (JNK1), MBL2, MPO, MX1, MYD88, NFKB1, NFKBIA (I?Ba/Mad3), STAT1, TICAM1 (TRIF), TRAF6. |
| --- |
| **Adaptive Immunity:**  Th1 Markers/Immune Response: CCR5, CD80, CXCR3, IFNG, IL18, IL23A, SLC11A1, STAT4, TBX21, TLR4, TLR6. Th2 Markers/Immune Response: CCR4, CCR8, CD86, GATA3, IFNB1, IL10, IL13, IL18, IL4, IL5, IL6, NOD2, STAT6. Th17 Markers: CCR6, IL17A, RORC, STAT3. Treg Markers: CCR4, CCR8, FOXP3, IL10. T Cell Activation: CD80, CD86, ICAM1, IFNG, IL23A, IL6, SLC11A1. Cytokines: CCL2 (MCP-1), CCL5 (RANTES), CSF2 (GM-CSF), CXCL10 (INP10), IFNA1, IFNG, IL10, IL13, IL17A, IL18, IL2, IL23A, IL4, IL5, IL6, IL8, TNF. Other Genes: CD4, CD40 (TNFRSF5), CD40LG (TNFSF5), CD8A, CRP, FASLG (TNFSF6), HLA-A, IFNAR1, IFNGR1, IL1B, IL1R1, IRF3, IRF7, ITGAM, JAK2, MAPK8 (JNK1), MBL2, MX1, NFKB1, RAG1, STAT1. |
| **Humoral Immunity:** C3, CCL2 (MCP-1), CCR6, CRP, IFNB1, IFNG, IL6, MBL2, NOD2, TNF. |
| **Inflammatory Response:** APCS, C3, CCL5 (RANTES), CRP, FOXP3, IL1A, IL1B, IL4, IL6, MBL2, STAT3, TNF. |
| **Defense Response to Bacteria:** IFNB1, IFNG, IL23A, IL6, LYZ, MBL2, MYD88, NOD1 (CARD4), NOD2, SLC11A1, TLR1, TLR3, TLR4, TLR6, TLR9, TNF. |
| **Defense Response to Viruses:** CD4, CD40 (TNFRSF5), CD86, CD8A, CXCL10 (INP10), DDX58 (RIG-I), HLA-A, IFNAR1, IFNB1, IL23A, IL6, IRF3, NLRP3, TICAM1 (TRIF), TLR3, TLR7, TLR8, TYK2. |

**Gene that expresses change is marked red.**

**Toll-like receptor signaling pathway PCR Array （b）**

| **Toll-Like Receptors:**CD180 (LY64), SIGIRR, TLR1, TLR2, TLR3, TLR4, TLR5, TLR6, TLR7, TLR8, TLR9, TLR10. |
| --- |
| **Pathogen-Specific Responses:**  Bacterial: CCL2 (MCP-1), CD14, CD180 (LY64), FOS, HRAS, IL10, IL12A, IL1B, IL6, IL8, IRAK1, HMGB1, HSPA1A (HSP70 1A), JUN, LTA (TNFB), LY86 (MD-1), LY96, NFKBIA (IKBA/MAD3), PTGS2 (COX2), RELA, RIPK2, TLR2, TLR4, TLR6, TNFRSF1A, TICAM1 (TRIF).  Viral: EIF2AK2 (PRKR), IFNB1, IFNG, IL12A, IL6, IRF3, PRKRA, RELA, TBK1, TLR3, TLR7, TLR8, TNF, TICAM1 (TRIF).  Fungal/Parasitic: CLEC4E, HRAS, HSPA1A (HSP70 1A), IL8, TLR2, TIRAP. |
| **TLR Signaling:**  Negative Regulation:SARM1, SIGIRR, TOLLIP.  TICAM1 (TRIF)-Dependent (MYD88-Independent): IRF3, MAP3K7 (TAK1), TAB1, NR2C2, PELI1, TBK1, TICAM2, TLR3, TLR4, TRAF6, TICAM1 (TRIF).  MYD88-Dependent: IRAK1, IRAK2, MAP3K7 (TAK1), TAB1, MYD88, NR2C2, TIRAP, TLR1, TLR10, TLR2, TLR4, TLR5, TLR6, TLR7, TLR8, TLR9, TRAF6. |
| **Downstream Pathways and Target Genes:**  NF?B Pathway:BTK, CASP8, CHUK (IKKa), ECSIT (SITPEC), FADD, IKBKB, IL10, IL1B, IRAK1, IRAK2, IRF3, LY96, MAP3K1 (MEKK), MAP3K7, MAP4K4, NFKB1, NFKB2, NFKBIA (IKBA/MAD3), NFKBIL1, NFRKB, PPARA, REL, RELA, TNF, TNFRSF1A, UBE2N.  JNK/p38 Pathway:ELK1, FOS, IL1B, JUN, MAP2K3 (MEK3), MAP2K4 (JNKK1), MAP3K1 (MEKK), MAP3K7, MAPK8 (JNK1), MAPK8IP3, TNF.  JAK/STAT Pathway: CCL2 (MCP-1), CSF2 (GM-CSF), IFNG, IL12A, IL2, IL6.  Interferon Regulatory Factor (IRF) Pathway:  CXCL10 (INP10), IFNA1, IFNB1, IFNG, IRF1, IRF3, TBK1.  Cytokine-Mediated Signaling Pathway: CCL2 (MCP-1), CSF3 (GCSF), IL1A, IL1B, IL6, IRAK1, IRAK2, RELA, SIGIRR, TNF, TNFRSF1A. |
| **Regulation of Adaptive Immunity:**CD80, CD86, HSPD1, IFNG, IL10, IL12A, IL1B, IL2, MAP3K7, TRAF6. |
| **Adaptors & TLR Interacting Proteins:**BTK, CD14, HMGB1, HRAS, HSPA1A (HSP70 1A), HSPD1, LY86 (MD-1), LY96 (MD-2), MAPK8IP3, MYD88, PELI1, RIPK2, SARM1, TICAM1 (TRIF), TICAM2 (TRAM), TIRAP, TOLLIP. |
| **Effectors:**CASP8 (FLICE), EIF2AK2 (PRKR), FADD, IRAK1, IRAK2, IRAK4, MAP3K7 (TAK1), TAB1, NR2C2, PPARA, PRKRA, ECSIT (SITPEC), TRAF6, UBE2N. |

**Gene that expresses change is marked red.**

**RT² Profiler PCR Array Human Tissue Innate & Adaptive Immune Responses Pathway(c)**

**(QIAGEN, Cat. no. PAHS-052Z)**

| **Position** | **Gene Symbol** | **Fold Regulation** | **Comments** | **RT²Catalog #** |
| --- | --- | --- | --- | --- |
| B07 | CXCL10 | 3.02 | A | [PPH00765E](https://geneglobe.qiagen.com/search?cat&amp;q=PPH00765E) |
| F01 | NLRP3 | 2.39 |  | [PPH13170A](https://geneglobe.qiagen.com/search?cat&amp;q=PPH13170A) |
| C07 | IFNG | -2.39 | B | [PPH00380C](https://geneglobe.qiagen.com/search?cat&amp;q=PPH00380C) |
| C10 | IL13 | -2.03 | B | [PPH00688F](https://geneglobe.qiagen.com/search?cat&amp;q=PPH00688F) |
| D03 | IL1R1 | -2.22 |  | [PPH00274A](https://geneglobe.qiagen.com/search?cat&amp;q=PPH00274A) |
| E08 | MPO | -2.10 | B | [PPH06082F](https://geneglobe.qiagen.com/search?cat&amp;q=PPH06082F) |
| H03 | GAPDH | -2.27 |  | [PPH00150F](https://geneglobe.qiagen.com/search?cat&amp;q=PPH00150F) |

**HSIL vs NC**

**CSCC** vs **NC**

| **Position** | **Gene Symbol** | **Fold Regulation** | **Comments** | **RT² Catalog #** |
| --- | --- | --- | --- | --- |
| B02 | CD80 | 3.02 | B | [PPH00860F](https://geneglobe.qiagen.com/search?cat&amp;q=PPH00860F) |
| B03 | CD86 | 2.12 | A | [PPH00826A](https://geneglobe.qiagen.com/search?cat&amp;q=PPH00826A) |
| B07 | CXCL10 | 35.35 | A | [PPH00765E](https://geneglobe.qiagen.com/search?cat&amp;q=PPH00765E) |
| C03 | ICAM1 | 2.32 |  | [PPH00640F](https://geneglobe.qiagen.com/search?cat&amp;q=PPH00640F) |
| D01 | IL1A | 3.94 | B | [PPH00690A](https://geneglobe.qiagen.com/search?cat&amp;q=PPH00690A) |
| D08 | IL6 | 3.27 | A | [PPH00560C](https://geneglobe.qiagen.com/search?cat&amp;q=PPH00560C) |
| D09 | CXCL8 | 3.89 |  | [PPH00568A](https://geneglobe.qiagen.com/search?cat&amp;q=PPH00568A) |
| E09 | MX1 | 2.20 |  | [PPH01325A](https://geneglobe.qiagen.com/search?cat&amp;q=PPH01325A) |
| F07 | STAT1 | 2.93 |  | [PPH00811C](https://geneglobe.qiagen.com/search?cat&amp;q=PPH00811C) |
| H04 | HPRT1 | 2.22 |  | [PPH01018C](https://geneglobe.qiagen.com/search?cat&amp;q=PPH01018C) |
| A02 | C3 | -7.09 |  | [PPH01185E](https://geneglobe.qiagen.com/search?cat&amp;q=PPH01185E) |
| B01 | CD40LG | -2.12 | B | [PPH00259C](https://geneglobe.qiagen.com/search?cat&amp;q=PPH00259C) |
| D03 | IL1R1 | -7.48 |  | [PPH00274A](https://geneglobe.qiagen.com/search?cat&amp;q=PPH00274A) |
| F04 | RAG1 | -3.40 |  | [PPH09892A](https://geneglobe.qiagen.com/search?cat&amp;q=PPH09892A) |
| F05 | RORC | -5.69 |  | [PPH05877A](https://geneglobe.qiagen.com/search?cat&amp;q=PPH05877A) |
| G05 | TLR5 | -2.36 | A | [PPH01793F](https://geneglobe.qiagen.com/search?cat&amp;q=PPH01793F) |
| G11 | TRAF6 | -2.60 |  | [PPH00329B](https://geneglobe.qiagen.com/search?cat&amp;q=PPH00329B) |
| H03 | GAPDH | -3.76 |  | [PPH00150F](https://geneglobe.qiagen.com/search?cat&amp;q=PPH00150F) |

**RT² Profiler PCR Array Human Tissue Toll-Like Receptor Signaling Pathway(d)**

**(QIAGEN, Cat. no. PAHS-018Z)**

**HSIL vs NC**

| **Position** | **Gene Symbol** | **Fold Regulation** | **Comments** | **RT² Catalog #** |
| --- | --- | --- | --- | --- |
| A12 | CXCL10 | 4.33 | A | [PPH00765E](https://geneglobe.qiagen.com/search?cat&amp;q=PPH00765E) |
| A09 | CLEC4E | -2.12 | B | [PPH06040A](https://geneglobe.qiagen.com/search?cat&amp;q=PPH06040A) |
| G07 | TLR9 | -3.12 |  | [PPH01809A](https://geneglobe.qiagen.com/search?cat&amp;q=PPH01809A) |

**CSCC** vs **NC**

| **Position** | **Gene Symbol** | **Fold Regulation** | **Comments** | **RT²Catalog #** |
| --- | --- | --- | --- | --- |
| A06 | CD80 | 2.54 | B | [PPH00860F](https://geneglobe.qiagen.com/search?cat&amp;q=PPH00860F) |
| A12 | CXCL10 | 47.42 | A | [PPH00765E](https://geneglobe.qiagen.com/search?cat&amp;q=PPH00765E) |
| B12 | IFNG | 4.05 | B | [PPH00380C](https://geneglobe.qiagen.com/search?cat&amp;q=PPH00380C) |
| C04 | IL1A | 2.22 | B | [PPH00690A](https://geneglobe.qiagen.com/search?cat&amp;q=PPH00690A) |
| C07 | IL6 | 4.40 | A | [PPH00560C](https://geneglobe.qiagen.com/search?cat&amp;q=PPH00560C) |
| C08 | CXCL8 | 4.61 |  | [PPH00568A](https://geneglobe.qiagen.com/search?cat&amp;q=PPH00568A) |
| C12 | IRF1 | 2.10 |  | [PPH00320F](https://geneglobe.qiagen.com/search?cat&amp;q=PPH00320F) |
| A05 | CD180 | -2.17 | B | [PPH06054A](https://geneglobe.qiagen.com/search?cat&amp;q=PPH06054A) |
| A09 | CLEC4E | -6.01 |  | [PPH06040A](https://geneglobe.qiagen.com/search?cat&amp;q=PPH06040A) |
| A11 | CSF3 | -2.94 |  | [PPH00723B](https://geneglobe.qiagen.com/search?cat&amp;q=PPH00723B) |
| E11 | PTGS2 | -3.94 |  | [PPH01136F](https://geneglobe.qiagen.com/search?cat&amp;q=PPH01136F) |
| F03 | SARM1 | -3.04 | B | [PPH06037A](https://geneglobe.qiagen.com/search?cat&amp;q=PPH06037A) |
| F04 | SIGIRR | -2.83 |  | [PPH06049A](https://geneglobe.qiagen.com/search?cat&amp;q=PPH06049A) |
| F08 | TICAM2 | -2.01 |  | [PPH06042A](https://geneglobe.qiagen.com/search?cat&amp;q=PPH06042A) |
| F09 | TIRAP | -3.85 |  | [PPH06246B](https://geneglobe.qiagen.com/search?cat&amp;q=PPH06246B) |
| G03 | TLR5 | -3.76 | A | [PPH01793F](https://geneglobe.qiagen.com/search?cat&amp;q=PPH01793F) |
| G07 | TLR9 | -2.88 |  | [PPH01809A](https://geneglobe.qiagen.com/search?cat&amp;q=PPH01809A) |
| G10 | TOLLIP | -2.58 |  | [PPH05844C](https://geneglobe.qiagen.com/search?cat&amp;q=PPH05844C) |
| G11 | TRAF6 | -2.13 |  | [PPH00329B](https://geneglobe.qiagen.com/search?cat&amp;q=PPH00329B) |
| H03 | GAPDH | -2.29 |  | [PPH00150F](https://geneglobe.qiagen.com/search?cat&amp;q=PPH00150F) |

**Supplementary Table S3**

E6 and E7 primers sequence

| HPV-16-E6 forward | CTGCAAGCAACAGTTACTGC |
| --- | --- |
| HPV-16-E6 antisense | GGCTTTTGACAGTTAATACACC |
| HPV-16 E7 forward | CATGGAGATACACCTACATTGC |
| HPV-16 E7 antisense | CACAACCGAAGCGTAGAGTC |
| GAPDH forward | GACAGTCAGCCGCATCTTCT |
| GAPDH antisense | TTAAAAGCAGCCCTGGTGAC |
